# A Personalized, Transdiagnostic Smartphone App (Mello) Targeting Repetitive Negative Thinking for Depression and Anxiety: Qualitative Analysis of Young People’s Experience

**DOI:** 10.2196/63732

**Published:** 2024-11-27

**Authors:** Lee Valentine, Chelsea Arnold, Jennifer Nicholas, Emily Castagnini, Jessi Malouf, Mario Alvarez-Jimenez, Imogen H Bell

**Affiliations:** 1 Orygen Melbourne Australia; 2 Orygen Parkville Australia

**Keywords:** repetitive negative thinking, rumination, anxiety, depression, mobile app, just-in-time adaptive interventions, youth mental health, adolescent, mobile phone

## Abstract

**Background:**

The increasing rates of mental health challenges among young people highlight an urgent need for accessible and effective treatment. However, current mental health systems face unprecedented demand, leaving most young people globally with unmet mental health needs. Smartphones present a promising solution to this issue by offering in-the-moment support through innovative just-in-time adaptive interventions, which provide support based on real-time data.

**Objective:**

This study explores young people’s experiences with Mello, a just-in-time adaptive intervention that focuses on the transdiagnostic mechanism of repetitive negative thinking (RNT), a significant factor contributing to youth depression and anxiety.

**Methods:**

Semistructured qualitative interviews were conducted with 15 participants aged 16 to 25 years, all of whom had previously participated in a pilot randomized controlled trial of Mello. Of the 15 participants, 9 (60%) identified as women, 4 (27%) as men (including 1 transgender man), and 2 (13%) as nonbinary. Interviews focused on participants’ experiences with the Mello app, factors influencing engagement, perceived benefits and limitations, and suggestions for future improvements. Thematic analysis was used to analyze the data.

**Results:**

The analysis identified three superordinate themes: Mello as a tool for intentional reflection; doing therapy your own way; and barriers to engagement during low mood, anxiety, and RNT. Theme 1 explored young people’s experiences of how the app facilitated active management of negative thoughts and supported the development of reflective habits, contrasting with their typical strategies of avoidance or resistance. Theme 2 highlighted the value of the app’s self-guided nature, with a particular focus on its flexibility and accessibility, particularly when compared to traditional face-to-face therapy. Finally, theme 3 addressed barriers to engagement, particularly during emotionally difficult times, with participants reporting feeling “stuck” in their negative thoughts. To mitigate these challenges, participants suggested incorporating gamification elements, such as progress-tracking visuals, to enhance motivation and increase engagement with the app.

**Conclusions:**

Our findings underscored the value of Mello in promoting intentional engagement and reflection with RNT, consistent with prior research that emphasizes the effectiveness of tailored interventions. Although some users valued the self-guided nature of the application, others encountered difficulties with motivation. Future research should explore strategies to enhance engagement for young people with low mood and motivation, such as co-design methodologies, advanced personalization features, and gamification techniques.

## Introduction

### Background

Young people are facing a crisis of mental ill-health, with estimates suggesting that 1 in 5 have a mental health disorder [1], making them the most disproportionately affected age group for mental health burden [2], with upward of 50% affected by the age of 25 years [3]. The magnitude of this problem has increased over the course of the global pandemic [4], and rates of mental ill-health have steadily escalated over the last decade, placing unprecedented demands on services that exceed available resources [3,5]. For example, an Australian survey of young people found 44.1% of the general population met the criteria for depression and 48.5% for anxiety [6]. Critically, estimates suggest that only 50% of young people with mental ill-health seek help [7]. Among those who do seek care, many encounter long waiting periods [8] and disengage prematurely, resulting in only a small proportion of people who experience long-term recovery [9,10]. Due to these barriers, as many as 55% of young people worldwide are experiencing unmet needs for mental health care [11]. Smartphone technologies present an important opportunity to address the growing burden of mental ill-health among young people by providing real-time support without reliance on traditional face-to-face services [12,13].

High rates of smartphone ownership, coupled with a strong interest in using digital devices for mental health support among young people and clinicians, provide important opportunities to overcome barriers to mental health care for youth [14]. Smartphones possess a critical capability for the self-management of mental ill-health, enabling the detection and response to daily life experiences through personalized interventions [15]. “Just-in-time adaptive interventions” (JITAIs) represent a novel approach wherein smartphones or other mobile devices are leveraged to tailor momentary intervention strategies based on an individual’s specific context and needs [16]. While research evidence is still developing, a meta-analysis of 33 studies spanning physical and mental health conditions found evidence supporting the effectiveness of JITAIs compared to controls [17].

A unique opportunity to improve treatments for depression and anxiety in young people may involve the use of JITAIs to deliver momentary interventions targeting transdiagnostic mechanisms in real-time, real-world scenarios. Transdiagnostic mechanisms refer to underlying psychological or behavioral processes that contribute to multiple mental health conditions irrespective of their specific diagnosis [18]. These mechanisms potentially offer a more personalized and precise intervention target, applicable across a diverse array of mental health disorders, thereby enhancing their relevance and efficacy [19]. JITAIs vary in their complexity, from basic static algorithms that select a limited number of interventions to more sophisticated systems that are contextually responsive and capable of learning from user data and individual behavior. However, many JITAIs currently in use rely on static algorithms [20]. Repetitive negative thinking (RNT; [21]) is a putative transdiagnostic mechanism with considerable evidence implicating a causal role in driving depression and anxiety symptoms, making it a highly promising intervention target [22]. RNT involves frequently occurring negative thoughts that are passive or hard to control, such as worry (concern for future events) and rumination (concern for past or current events or depressive symptoms; [21]). High levels of RNT are commonly observed among clinical populations of youth with clinical depression and anxiety and are highly correlated with the severity of depression and anxiety symptoms [23]. Inducing RNT in experimental studies results in worsening mood states [24], and heightened RNT predicts the later onset of emotional disorders in youth [25,26]. Meta-analyses have further shown that improvements in RNT are associated with reductions in depression and anxiety following psychological treatment in adults [27,28] and youth [14]. Therefore, targeting a reduction in RNT in treatment appears to be an important component of psychological treatment.

Various psychological treatment approaches, such as rumination-focused cognitive behavioral therapy, metacognitive therapy, and attention training, have been found to improve RNT outcomes [5]. However, traditional treatment delivery methods involving infrequent face-to-face sessions with a clinician suffer not only limitations in accessibility but are also poorly timed to target the process of RNT as it occurs in daily life. RNT is a dynamic process that is influenced by external and internal events over time [29]. Therefore, intervention strategies should be appropriately timed and sensitive to contextual variables that might influence how this process unfolds over time [30]. JITAIs are potentially well suited to targeting RNT in real time by detecting and delivering highly personalized and context-sensitive interventions designed to disrupt the process as it is happening. To test this theoretical intervention model, a JITAI called Mello was developed.

Mello is a self-guided smartphone intervention designed to reduce RNT (referred to as “stuck thinking” within the app) among young people experiencing depression and anxiety [31]. By using momentary assessments of stuck thinking intensity, mood states, location, and activity, users receive tailored recommendations for a microintervention strategy aimed at disrupting RNT in real time. In a pilot randomized controlled trial (RCT) [31] involving young people who used Mello for 6 weeks compared to a nonactive control group (n=55), the intervention was found to be acceptable, feasible, and potentially effective at reducing RNT, leading to improvements in depression and anxiety with moderate to large effect sizes. Furthermore, 96% of young people would recommend the app to others with similar experiences, with high satisfaction ratings across all domains. Young people also showed sustained use of Mello over time, with average use on 52% of total days and 59% of young people engaging with the app for the full 6-week period. These results demonstrated the highly promising potential of the Mello app and the broader theoretical concept of harnessing JITAs to target transdiagnostic mechanisms using personalized, context-sensitive interventions within real-time, real-world environments.

### Objectives

Building on the results of the RCT evaluating the feasibility and preliminary treatment effects of the Mello app [31], this study aimed to qualitatively explore young people’s experiences with Mello. This qualitative perspective can provide rich insights into how young people engage with and make meaning of their use of Mello, perceived benefits and limitations, and suggestions for refining and enhancing future iterations of the app. A better understanding of young people’s experiences with Mello not only informs potential benefits and areas for improvement but also contributes to the broader understanding of designing mobile real-time digital mental health interventions to enhance outcomes for young people experiencing mental health challenges.

## Methods

### Setting

This qualitative study was conducted as part of a broader research project examining Mello—a transdiagnostic smartphone app targeting RNT for young people experiencing anxiety or depression. The feasibility, acceptability, and preliminary clinical outcomes and mechanisms of Mello were examined in a pilot RCT [31]. During the pilot, participants were randomly assigned to either Mello or a nonactive control group for a 6-week period. This qualitative study was conducted with a subset of participants of the Mello intervention group willing to be interviewed following the completion of the 6-week intervention period.

**Figure 1:**
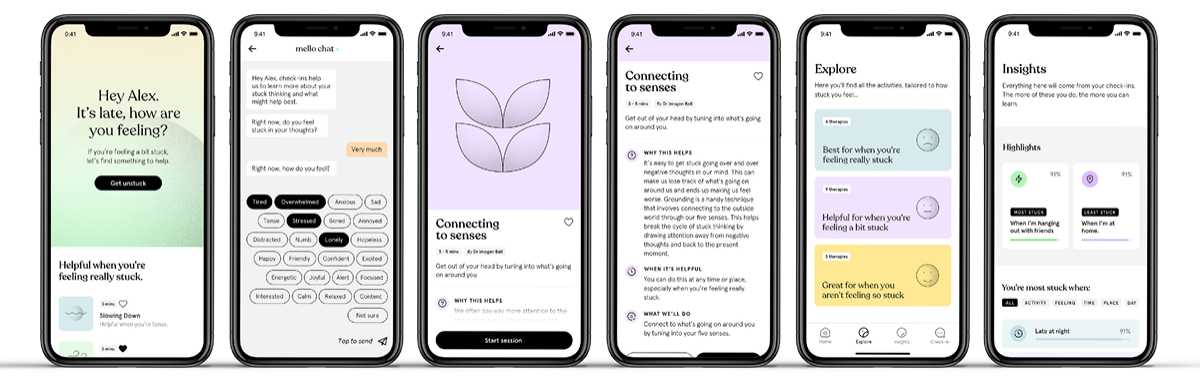
Mello app features.

Mello’s use of the JITAI model involved identifying and intervening in moments of need by using real-time assessments to tailor momentary intervention strategies aimed at disrupting RNT (Figure 1). In the pilot RCT, participants allocated to the Mello group received randomized notifications 3 times a day prompting them to complete “check-ins”: a brief 4-item questionnaire completed via the chat interface assessing the level of RNT, mood, current activities, and location. On the basis of the response provided during the check-ins, the app recommended 1 of 12 cognitive behavioral activities (Multimedia Appendix 1). Each activity took approximately 2 to 12 minutes to complete and was delivered through guided text or audio instructions using evidence-based strategies for targeting RNT. The selection of therapeutic activities was informed by a meta-analysis of psychological treatment approaches for RNT [5]. These activities included exercises such as mindfulness, problem-solving, cognitive defusion, worry time, thought challenging, gratitude, and self-compassion [31].

### Sampling and Recruitment

Participants in the pilot RCT were aged 16 to 25 years, experiencing clinical levels of depression and anxiety (≥10 on the Patient Health Questionnaire 8 [32] or Generalized Anxiety Disorder Scale 7 [33]), and elevated RNT levels (≥37) on the Perseverative Thinking Questionnaire [21]. Additional inclusion criteria involved the ability to provide informed consent, sufficient command of the English language, and ownership of a smartphone capable of running the Mello app (Android [Google LLC] or iPhone [Apple Inc]). Exclusion criteria comprised current psychiatric inpatient status or receiving treatment from a mental health crisis management team. There were no additional exclusion or inclusion criteria, beyond being randomized to the Mello arm of the RCT, for participating in the present qualitative study.

All participants assigned to the Mello intervention arm of the pilot RCT (N=29) consented to follow-up after the 6-week intervention period and were invited to participate in a qualitative interview exploring their experience with Mello. As such, at the conclusion of the intervention period, emails were sent to all participants in the Mello intervention arm, inviting them to participate in a qualitative interview. Of the 29 young people invited, 15 (52%) responded and agreed to participate. The gender distribution, mean age, and country of birth within this subgroup were consistent with the overall sample from the pilot RCT.

There was variability in app engagement among the sample: with active use ranging from 6 to 38 days (out of a possible 42) during the 6-week intervention period (mean 21.9, SD 11.1) The engagement levels among this sample were reflective of the broader use patterns observed in the original trial, where participants used the app on 49.2% of days and completed an average of 29.9 check-ins [31]. This variation in engagement is valuable in qualitative research, as it allows a more nuanced understanding of user experiences across the spectrum of engagement, providing insights into both facilitators and barriers to sustained app use across different engagement levels. By including participants with varying degrees of interaction with Mello, this study aimed to identify the factors that either support or hinder ongoing engagement with the app, offering a more nuanced perspective on its efficacy and usability.

### Procedure

The qualitative interviews were conducted between February 8, 2022, and May 2, 2022. On average, participants were interviewed 1.9 weeks (range 0-4.7 wk) following the final date they had access to the Mello app. All interviews were conducted via Zoom (Zoom Video Communications, Inc) by authors CA and EC. CA, a female clinical psychologist with a PhD in clinical psychology, specializes in digital mental health and has extensive experience working clinically with people experiencing mental health challenges. She has led and contributed to multiple qualitative studies from study design to data collection and analysis. EC, also a female researcher, holds a master’s degree in public health and has substantial demonstrated experience in qualitative research, particularly in co-designing and interviewing young people about their experiences with digital mental health products. Both CA and EC had prior relationships with participants, as they were responsible for conducting pre and post-interviews with participants as part of the Mello RCT.

The interview schedule was designed to better understand young people’s experiences and perspectives of the Mello app. Multimedia Appendix 2 provides the full interview schedule. All interviews were conducted and audio recorded via Zoom to facilitate transcription and analysis. The recordings were transcribed verbatim by a third-party service. Both the audio files and transcripts were stored securely on a password-protected server. Identifying information, such as participant consent forms, was stored separately to ensure confidentiality. Access to the recorded interviews and transcripts was restricted to the immediate research team responsible for conducting the analysis.

### Data Analysis

Thematic analysis, guided by the framework proposed by Braun and Clarke [34], was used for data analysis. To begin, a single transcript was independently coded by authors JN, LV, CA, and IB. Following this, the 4 authors participated in collaborative discussions to review and compare their initially identified codes and coding process. This iterative process allowed for the refinement of an initial coding dictionary, resulting in the development of a robust coding framework.

To further ensure consistency, authors LV and CA independently coded and discussed 2 additional identical transcripts. This additional coding was compared to the established framework to further solidify alignment and consistency in the coding process. With confidence in the coding alignment, author LV proceeded to independently code the remaining 12 interviews. The coded data were then systematically grouped based on similarities, and through an iterative process among authors, overarching themes and subordinate themes were developed.

The systematic thematic analysis approach used in this study established a strong framework for comprehensively capturing and understanding the nuanced experiences and perspectives of participants using Mello.

### Ethical Considerations

The study was approved by the University of Melbourne Human Research ethics committee (project 2021-22524-23553-5). Participants provided written informed consent through the REDCap (Research Electronic Data Capture; Vanderbilt University) web application. Before providing consent, participants were given a thorough explanation of the study in simple language, including the study’s aims, procedures, and any potential risks or benefits. Participants were also reminded that they could withdraw from the study at any time, skip questions, or take a break from the interview without any negative consequences. Participants were given a copy of the participant information and consent form and received Aus $45 (US $29.5) as reimbursement for their participation in the research interview. All data were deidentified.

### Reflexivity

All research questions were intentionally designed to be open-ended to prevent leading participants in predetermined or unconsciously biased directions. In the interview consent process, young people were explicitly assured that their honest opinions were valued, and the research team welcomed negative and constructive feedback as much as positive input.

Throughout the analysis phase, the research team held regular meetings to critically review codes and thematic patterns. These sessions were instrumental in considering how the research teams’ personal experiences, viewpoints, beliefs, and biases may be shaping the interpretation of results and influencing data analysis. The team acknowledges the potential impact of their own beliefs and experiences on the interpretation process.

Ultimately, participants’ experiences were deeply considered, with the research team composing rich and detailed paragraphs linked to illustrative quotes. This approach aims to ensure a transparent and traceable lineage between the reported findings and the actual words and experiences shared by the young people themselves.

## Results

### Overview

The study included 15 participants, with ages ranging from 16 to 25 (mean 20.9, SD 2.9) years. Overall, 60% (9/15) of participants identified as women, 27% (4/15) as men (including 1 transgender man), and 13% (2/15) as nonbinary. Furthermore, 73% (11/15) of the participants were born in Australia, while 27% (4/15) were born overseas. In addition, 53% (9/15) of the sample were receiving mental health treatment while using Mello. The average interview duration was 31.9 minutes, with interview times ranging from 17.0 to 67.2 minutes. Additional demographic details are presented in Table 1.

The analysis identified three superordinate themes reflecting distinct aspects of participants’ experiences with the Mello app: (1) Mello as a tool for intentional reflection; (2) doing therapy your own way; and (3) barriers to engagement during moments of low mood, anxiety, and RNT. These themes, along with representative quotes from participants, are presented in the *Results* section.

Superordinate theme 1 highlighted how Mello served as a tool for intentional engagement with RNT. Participants described how the app facilitated active self-management of negative thoughts, contrasting with their usual strategies of either resisting support or avoiding difficult emotions. Subordinate themes include habit formation, where automated check-ins helped participants build consistent reflective habits, and enhanced self-awareness, allowing them to recognize emotional patterns through personalized reflection.

Superordinate theme 2 focused on the value of Mello’s self-guided nature, enabling participants to manage their mental health autonomously. Many participants found that the app’s flexibility and accessibility alleviated the anxiety that can be associated with traditional face-to-face therapy, and they appreciated its availability during late hours or when other support systems were inaccessible.

Superordinate theme 3 explored the barriers to engagement, particularly during moments of low mood, anxiety, and RNT. Some participants struggled to engage with the app during emotionally difficult times, feeling “stuck” in their negative thoughts. To address these challenges, participants suggested incorporating gamification elements, such as progress-tracking visuals, to enhance motivation and make the app more engaging and rewarding. Textbox 1 presents a visual representation of these themes.

**Table 1:** Demographic characteristics of participants.

Participant ID	Age (years)	Gender	Country of birth	Currently receiving treatment?	Total use days on Mello, n
1	20	Woman	Australia	Yes	32
2	20	Man	Australia	Yes	27
3	25	Man, transgender	Australia	No	28
4	20	Man	Australia	No	9
5	16	Woman	Australia	Yes	8
6	24	Woman	Australia	No	14
7	17	Nonbinary	Australia	Yes	33
8	20	Nonbinary	Australia	Yes	17
9	24	Woman	China	Yes	12
10	21	Woman	Bangladesh	No	32
11	24	Woman	Australia	No	6
12	25	Woman	Australia	Yes	38
13	17	Man	Australia	Yes	37
14	20	Woman	India	No	19
15	20	Woman	India	Yes	16

**Superordinate and subordinate themes.**

**Mello as a tool for intentional engagement with repetitive negative thinking (RNT)**

- Habit formation
- Enhanced self-awareness through personalized reflection and pattern recognition

**Doing therapy your own way**

- The value of self-guided therapy
- Accessibility and dependability

**Barriers to engagement during moments of low mood, anxiety, and RNT**

- Avoiding difficult feelings and getting “stuck” in RNT
- Leveraging gamification to enhance self-guided motivation

### Superordinate Theme 1: Mello as a Tool for Intentional Engagement With RNT

#### Overview

Participants commonly characterized Mello as a tool that facilitated intentional engagement with their thoughts and feelings. A participant noted the following:

It was really nice to just take a moment and figure it out and maybe work through a little bit of anxiety or something that I wasn’t quite recognising in the moment.

Participant 8

Several participants identified that this deliberate approach to *thinking,* sharply contrasted with their typical responses to rumination. Young people generally described these established responses in two distinct ways: resisting support and persisting in a state of rumination, or attempting to avoid engaging with their thoughts altogether. One participant described the impulse to resist support, stating:

I feel an urge to remain in that state and to continue ruminating or feeling bad and therefore a resistance to access any kind of assistance that could help me.

Participant 2

In contrast, another participant shared the belief that avoiding reflection on RNT might make distressing thoughts disappear, although this often had the opposite effect:

Sometimes you feel like you should just avoid it entirely. Maybe if I just avoid reflecting on it, then it’ll go away or something, and it obviously doesn’t, it sometimes makes it worse.

Participant 10

In contrast to these typical responses to rumination, young people described using Mello as a means for actively managing negative thoughts through deliberate reflection. This approach helped them to diverge from habitual responses, like avoidance, and empowered them to actively self-manage negative thoughts in the moment. One participant explained:

Once the negative thoughts come on, I would immediately go to the app, and then that way I would kind of help myself sort it out through that.

Participant 3

Beyond seeking relief during “stuck” moments, some young people expressed a proactive desire to practice skills during moments when they were not feeling “stuck,” using their clearer frame of mind, aiming to better equip themselves for difficult moments in the future. One participant shared:

And one of the options [is] that people use it or practice the activities that are useful when you are stuck specifically when they are not so that they gradually feel more comfortable trying those activities when being least stuck to most stuck as they feel more and more stuck. Now they feel comfortable doing those activities. As familiarity increases, the range they feel comfortable doing them increases in moments of stuckness.

Participant 2

This proactive approach demonstrates young people’s creative use of Mello to practice skills and build resilience during moments of calm, a valuable way for young people to prepare for and manage future challenges with RNT more effectively.

#### Subordinate Theme 1.1: Habit Formation

Participants commented frequently on Mello’s automated check-ins, emphasizing their role in prompting reflection on thoughts, feelings, and surroundings. For some, these *check-ins* felt “difficult*,*” particularly when: (1) the young person was experiencing low mood or motivation and was concerned that engaging with Mello would exacerbate these feelings, or (2) the young person felt that their “lack of energy” from low mood, stress, or anxiety was too limited to engage with the process.

For others, the check-ins served as a functional “trigger,” or practical reminder, helping to establish a daily “habit” of self-reflection. Participant 9 framed these check-ins as an instructional cue to “review feelings.” Several participants noted that regularly using Mello helped them build a greater sense of self-awareness of their thoughts and emotions. A participant shared:

It was really helpful in building my awareness of how I felt at different times during the day when I did do it consistently and tying that to different activities, or people I was around.

Participant 2

For some young people, the check-ins were experienced as thoughtful gestures, conveying a sense that someone was thinking of them and cared about them. A participant expressed the following:

Every time I see the notice from Mello...[it] gives me a kind of feeling, like friends, they really care about you.[Participant 9]

This reminder encouraged some young people to remember to extend that sense of care toward themselves. One participant described the experience as follows:

So, it’s actually quite wholesome to be like, “Hey, how you going?” It’s just nice to have that little reminder. It’s like, “Hey.” And it reminds you, “Oh, I need to like think about myself as well.”[Participant 3]

These insights highlight the meaningful impact of Mello’s check-ins, serving not only as a reminder prompting self-reflection but also as a source of perceived care and support. This contributing to a positive and “wholesome” experience akin to the thoughtful gesture of friends checking in on each other.

However, while some identified the check-ins as helpful stepping stones toward building a positive habit of reflection, others acknowledged the difficulty they had in forming such habits. To better support this process, participants suggested that Mello could make the check-in process more compelling and “attractive” thereby increasing the likelihood that a young person would engage and build the habit over time. Participant 2 recommended leveraging the “habit loop of cue and craving routine reward*,*” suggesting that making the cue obvious, the craving appealing, the routine easy, and the reward more satisfying could increase the likelihood of engagement. Furthermore, Participant 2 proposed that notifications could prompt the user to “imagine” the positive feelings they would experience in the future after checking in or completing a therapy activity, effectively linking the cue to the satisfaction of the craving.

Despite young people’s general support for the notifications and regular check-ins, participants emphasized the need for customizable notification schedules to align with various life commitments, such as work, social engagements, and family obligations. This customization also enables users to safeguard their own personal free time without interruption. These examples highlight the importance of a more personalized experience with Mello, ensuring a more seamless integration into the intricacies of daily life.

#### Subordinate Theme 1.2: Enhanced Self-Awareness Through Personalized Reflection and Pattern Recognition

While some participants found checking in with Mello to be a valuable act of self-reflection of its own accord, others described it as a tool for gaining a deeper understanding of the connection between their “feelings and patterns of behaviour,” thereby enhancing their sense of self-awareness. By creating moments for self-reflection, Mello supported young people to recognize emotions, such as anxiety or anger, that might have otherwise gone unnoticed and potentially intensified. One likened this experience to mindfulness:

It almost felt like mindfulness, acknowledging that I’m in my room, alone, and feeling angry. I found that really positive.

Participant 2

Some users reported that regularly using the Mello app to track their thoughts, emotions, and surroundings helped them to identify recurring emotional and behavioral patterns. This data-driven approach motivated them to stay engaged with the app throughout the intervention period. Recognizing personal patterns, particularly in relation to daily activities and specific times of day, was considered valuable. One participant highlighted the importance of immediate access to personal data via Mello:

Being able to notice my own patterns, especially when it comes to the activities I’m doing and times of day, that was very important to me. The idea of having the statistics right there was motivating.

Participant 7

Participants commonly requested the ability to personalize the information recorded, including details about their environment, to use the app more effectively. They emphasized the need for this feature in order to better make informed choices about their mental health, based on accurately collected data, stressing the importance of clear and informative data that visually illustrated progress and discernible patterns over time.

### Superordinate Theme 2: Doing Therapy Your Own Way

#### Overview

A subgroup of young people consistently underscored two key aspects of using Mello: it’s self-guided nature and its accessibility and dependability. For some, Mello’s self-guided approach alleviated the anxiety typically associated with traditional face-to-face therapy. For others, the apps consistent support and constant availability was empowering, contributing positively to their mental health journey.

#### Subordinate Theme 2.1: The Value of Self-Guided Therapy

Some young people described experiencing the self-guided nature of Mello, which did not include a real-time face-to-face therapist, to be a feature that alleviated the anxiety typically associated with traditional face-to-face therapy dynamic, while also enhancing their sense of agency and control over their mental health treatment:

I think it was an interesting experience for me because I’ve never used an app before to manage my anxiety. And I think it was good to have that new technology experience that I hadn’t had before, because I’m so used to talking to people in person. And sometimes that has increased my anxiety and not made it even better so having an app was a nice change and a good experience for me.

Participant 7

This quote reflects the perspective of a subgroup of participants who saw Mello as an intervention providing valuable control and autonomy over their mental health experience. Participant 11 further described how they could lose sight of their own priorities in face-to-face therapy: “Sometimes other people’s input makes those anxious thoughts spiral, because I’m trying to fit someone else’s ideas and someone else’s intentions into my life and then I’m not prioritizing my own*.*” In this case, Mello offered a therapeutic avenue for identifying and addressing personal mental health priorities without inadvertently conforming to others’ expectations.

Furthermore, participant 11 appreciated the ability to work at their own pace, free from the perceived pressure of being “on someone else’s time*.*” They explained:

I liked that I could do it at my own pace. What I found before in doing psychology sessions in person is that I feel rushed and I feel like they’re expecting, even though it’s not the truth, but they’re expecting me to say something in a certain way. So having an app was a good change.

Participant 11

Participants described valuing the self-guided nature of Mello as it provided a nonjudgmental space to express their feelings. Participant 11 reflected on the positive experience of “venting of the feelings” by recording notes via microphone on the Mello app:

Yeah. Yeah. It kind of just made it feel like I have that freedom to say whatever I want and no one’s going to judge me for it or force me to act on something I don’t want to act on. Sometimes it’s just getting that information out there and that’s all you need to do rather than have to put together a whole strategy or plan out your entire week or whatever. It’s just getting it out there in that moment and then it’s done.

Participant 11

For some, Mello was experienced as a positive alternative to the traditional face-to-face therapeutic experience, allowing them to communicate their thoughts and emotions without the anxiety brought on by in-person interactions.

It was convenient in an app for me and that I could put in my answers and learn more about the different techniques to manage my anxiety. And yeah, it was just good that I had something respond to me and not feeling embarrassed or ashamed or just awkward.

Participant 11

The self-guided and autonomous nature of Mello empowered some participants to engage with the intervention components that most resonated with them. This flexibility enabled them to explore therapeutic content without being confined to a specific approach. One participant describes appreciating this approach:

I think the variety, there was lots of good variety in the different activities. And again, I really liked how you could both read it or listen to it. I really liked there was that option.

Participant 1

While preferences for therapeutic activities varied among participants, some highlighted the app’s capacity for positive distraction. For example, participant 9 shared, “I pay attention to the using, so the sadness feeling decreased while I use it*.*” Gratitude practices emerged as a gentle and affirming way for young people to engage with therapy, with one participant noting the following:

For example, the gratitude exercise where I had to put in effort into thinking about what was something positive in my day. Even if it was small, I realised that not everything in my day was crappy, and that’s great. Maybe it’s a small thing now, but tomorrow it’s going to be great, a bigger thing.

Participant 10

Overall, these diverse experiences highlighted how Mello’s self-guided approach, offering a variety of activities, allowed for individual choice and meaning-making.

#### Subordinate Theme 2.2: Accessibility and Dependability

Young people emphasized the benefit of having access to reliable, “24/7” mental health support through Mello. They particularly highlighted the app’s “availability,” and the perceived *“*trustworthiness” of the content. Participants commonly described turning to Mello when friends and family were unavailable, using it before bed, or in the later hours of the night and early morning when it was challenging to find support elsewhere. For example, participant 3 explained:

You can’t always go to your family members, and I mean you can, but I felt like I couldn’t, because I didn’t want to bring up anything bad for them. So, that’s where Mello was quite helpful, where I could just rant.

Participant 3

This high level of accessibility ensured that young people could rely on Mello for assistance whenever needed, solidifying the app’s value as a dependable self-guided resource.

Moreover, Mello’s continuous availability and support empowered young people to maintain autonomy in self-managing challenging experiences. One participant shared the following:

I think it just ultimately comes down to having an accessible resource so I don’t have to keep searching for anxiety relieving gifs online, it’s right there in the app for me, and I can just reach out for it. And yeah, I just really liked that.

Participant 10

### Superordinate Theme 3: Barriers to Engagement During Moments of Low Mood, Anxiety, and RNT

#### Overview

Some young people described experiences that hindered their use of Mello during moments of RNT. These experiences revolved around the perception that engaging with the app during moments of stuck thinking introduced an additional layer of difficulty to an already emotionally challenging time.

For some, feelings of distress or depression left them with limited energy, motivation, or desire to engage with Mello. Participant 4 noted, “But I think that just came down to general procrastination, forgetfulness, and fatigue because I’d feel tired a lot*.*”

Others hesitated to engage with their RNT altogether, fearing that doing so would worsen their emotions and exacerbate their feelings of distress. In this context, it was perceived that using the app had the potential to intensify their negative thinking patterns instead of providing relief, thereby contributing to the very feelings they were seeking to alleviate. Despite the potential for a positive outcome through engagement with the app and therapeutic guidance, this process felt too burdensome for some young people to endure.

#### Subordinate Theme 3.1: Avoiding Difficult Feelings and Getting “Stuck” in RNT

Several young people expressed a desire to avoid intentionally engaging with their difficult feelings and thoughts, believing that ignoring them would cause the discomfort to dissipate and “go away” of its own accord.

For some, purposefully engaging with their negative thoughts contradicted their established coping mechanisms, which typically involved attempting to avoid them. From this perspective, paying greater attention to their negative thoughts risked amplifying the negative feelings or prolonging the duration of their experience, both of which were outcomes they sought to avoid. One participant explained the following:

I see a notification telling me it’s time to check in with Mello. And I would dismiss that because it seems difficult, or I don’t want to confront how I’m feeling, because that could be painful if I’m already avoiding emotional and mental pain.

Participant 2

Thus, when prompted by Mello to check in, participant 2 dismissed the notification because engaging with their feelings seemed daunting and potentially distressing. This experience underscores a reluctance to confront emotional and mental pain, preferring instead to evade such experiences.

The concept of feeling “stuck” was a reoccurring theme among young people when describing their experiences of RNT. This term was also commonly used by young people when describing the impediments they experienced when trying to engage with Mello during moments of worry or anxiety. Many participants expressed that during moments of rumination, feeling “stuck” extended beyond their thought patterns and interfered with their behavior. This created a catch-22 situation, wherein the more entrenched in their thinking they became, the more challenging it became to actively participate in the intervention. One participant articulated this challenge, stating the following:

For me, if I am depression and the situation is really serious, I didn’t want to do anything else and I didn’t care about the app. I didn’t care anybody say anything. I just cannot get away that kind of feeling.

Participant 9

Consequently, struggling to engage with Mello while caught in cycles of RNT posed a formidable challenge to some young people. In general, young people reported that experiences of depression, distress, stress, and various life events, combined with the perceived emotional effort required to engage with Mello during moments of rumination, acted as barriers to use. Participant 2 explains, “There were times when it would pop up on my notifications, but I just didn’t have the energy to even express what I was feeling.”

#### Subordinate Theme 3.2: Leveraging Gamification to Enhance Self-Guided Motivation

Among participants, a distinction emerged between those who valued the autonomy inherent in the self-directed nature of the Mello app and those who found the need for self-motivation to be a barrier to sustained engagement. While some participants appreciated the flexibility to engage with the app at their own pace, others expressed difficulty in maintaining regular use without the external prompts or active follow-up typically provided by a service or clinician. This challenge of self-motivation was a common theme, with several participants emphasizing that without consistent guidance or reminders, it would be harder to prioritize the app as part of their daily life.

To address this, some users suggested that incorporating gamification elements, such as points, rewards, or progress-tracking visuals, could enhance their motivation to use the app. One participant highlighted the potential of integrating a growth-based visual representation, similar to an app they had used in high school, where a tree grows as the user studies or engages in tasks. Participant 2 explained the following:

I know that can be very scary for some people—they don’t want to be, oh, why is this app demanding me to use it? I don’t like that. But, I think when I used to be in high school, there was an app where you put in for how long you want to study for, and then it sets the timer and in that timer, a tree grows. And so, if you sort of kind of procrastinate within that period, that tree dies. Obviously, that wouldn’t be the case here, but I think if you sort of have a section where you’re, the more you use the app, the more the tree grows, it’s sort of you get to see a nice visual of you growing your skills and growing in your mental health journey and progress. I think that would’ve been great to see because it’s great to see these insights and all, but I want to see how am I also growing as a person who’s using these activities.

This suggestion underscores the importance of tangible feedback for young people, particularly those who struggle with self-motivation. For these participants, the ability to visually track their progress or achievements over time could enhance engagement by adding a layer of reward and satisfaction to the process of working on their mental health. Such features could not only enhance motivation but also provide users with a sense of accomplishment and progression, making the self-directed nature of the intervention feel more interactive and supportive.

## Discussion

### Principal Findings

This study aimed to explore young people’s experience of using Mello, a transdiagnostic intervention designed to alleviate RNT associated with depression and anxiety. A total of 15 participants took part in the study following a 6-week intervention period.

The analysis identified three key themes from participants’ experiences with the Mello app: (1) Mello as a tool for intentional reflection; (2) doing therapy your own way; and (3) barriers to engagement during low mood, anxiety, and RNT. In theme 1, participants described how the app could facilitate the active management of negative thoughts and supported the development of reflective habits, contrasting with their typical strategies of avoidance or resistance. Theme 2 highlighted the value of the app’s self-guided nature, with a particular focus on its flexibility and accessibility, particularly when compared to traditional face-to-face therapy. Finally, theme 3 addressed the barriers to engagement, particularly during emotionally difficult times, with participants reporting feeling “stuck” in their negative thoughts. To mitigate these challenges, participants suggested incorporating gamification elements, such as progress-tracking visuals, to enhance motivation and increase engagement with the intervention. Young people’s qualitative experiences spoke to the effectiveness of the JITAI framework [16] in fostering intentional engagement and reflection with thoughts and emotions in times of need, as presented in the first overarching theme. For some, this framework facilitated positive behavioral changes by encouraging active engagement with and reflection on their RNT. It offered a constructive alternative to habitual responses such as resisting support or attempting to avoid engaging with their rumination, with the hope of achieving quicker resolution from difficult feelings. Instead, it encouraged intentional engagement in real time, facilitated meaning-making, and, for some, led to a reduction in RNT. These results may provide explanations for the findings of the pilot clinical trial of Mello [31], which demonstrated moderate to large improvements in depression, anxiety, and RNT in Mello users versus the control group. This suggests that providing brief, smartphone-delivered interventions during moments of RNT offers a promising pathway for disrupting key processes in real time in a way that intentionally engages conscious behaviors and counteracts habitual maladaptive responses. This opportunity is uniquely suited to the JITAI framework and opens opportunities for future research to explore the application of this approach for targeting other transdiagnostic processes with temporal and contextual qualities, for example, emotion regulation, avoidance, and cognitive biases. Future research may benefit from identifying and refining the timing of intervention prompts to optimally disrupt the RNT process, as well as determining which intervention strategy may work best for the individual in any given moment. This can be achieved using artificial intelligence (AI) approaches, such as reinforcement learning [35], which can learn from individual user data to adapt both the timing and type of intervention over time. By understanding the variety of activities and intervention types chosen by the user, these AI systems can better enhance personalized recommendations and potentially improve outcomes. Our data support the efficacy of the JITAI approach in addressing RNT. We aim to advance from a basic JITAI 1.0 model to a more sophisticated intervention. This next-generation JITAI will incorporate a greater variety of activities and use contextual information and inputs, leveraging AI to tailor the timing and delivery of interventions. This enhancement is expected to significantly improve personalization and therapeutic outcomes.

These findings are consistent with meta-analytic review by Wang and Miller [17], which found that JITAIs saw an increased additive effect when tailored to the individuals’ behavioral patterns and current needs. Wang and Miller [17] also found that the effectiveness of the JITAIs was further enhanced by the integration of both a human agent and an algorithm to generate personalized feedback. This mirrors the approach implemented in the Mello intervention in which a static algorithm tailored intervention content specific to the needs and behaviors of the participants. These findings provide support for the effectiveness of tailoring interventions to individuals’ needs in the moment and underscore the importance of combining human and algorithmic support in digital interventions. While Mello is a self-guided app without human support, the current findings suggest future iterations should emphasize personalization. By incorporating personalized notifications and content recommendations, the app can customize the user experience, creating a more engaging and tailored environment that mimics the experience of human support [36].

While some young people acknowledged the benefits of using Mello during difficult moments of rumination, others had difficulty engaging with the app due to low mood, stress, the challenge of RNT, or lack of active follow-up due to the self-guided nature of the intervention, as detailed in theme 3. Challenges with motivation are a qualitatively well-documented barrier to engagement with mental health interventions, which may be particularly notable during periods of low mood, heightened anxiety, or with self-guided interventions [37-41]. This theme aligns with broader findings in the digital mental health literature that demonstrates maintaining engagement, particularly in real-world contexts, remains a considerable challenge, with many interventions experiencing low rates of sustained use [42-45]. This highlights the importance of JITAI’s and future iterations of Mello, which are designed to provide support in the moment and are well-positioned to engage people with personalized reminders and content that is immediately responsive to the thoughts and feelings people are experiencing in the moment.

Advancing digital therapeutic interventions to support mental health likely necessitates further research into the experience of people with low motivation and the development of strategies tailored for this group. One avenue for investigation could involve identifying effective techniques or features within digital interventions specifically designed to engage low- users with low motivation, such as personalized support and encouragement and reminders or motivational messages. Qualitative research by Berry et al [46] with mental health service users emphasized the importance of interventions being perceived as “positive, fun, practical and interactive,” underscoring the necessity for prioritizing engaging and user-centered design in digital mental health interventions to more effectively engage people, particularly those with low motivation, in managing their mental health.

Furthermore, co-design in the mental health context and the involvement of end users in the design of interventions have been associated with a generally enhanced effectiveness [47]. Therefore, actively engaging users with low motivation in the design and implementation process holds great promise for gaining valuable insights into their unique experience of low motivation and for effective solutions. Personalization, in particular, tailors the app’s features and content to align with the individual preferences and needs of each user, creating a more relevant, autonomous, and engaging experience [36,48]. Gamification introduces game-like elements and incentives to make interacting with the app more compelling and motivating [36]. In a 2024 study, Lieder et al [49] examined the optimization of gamification as a method to facilitate positive behavior change; for instance, in an app aimed at increasing water intake, users who received feedback based on optimized gamification were more likely to enact the desired behavior more frequently compared to control groups. Overall, further research in this area can help identify strategies to motivate individuals more effectively during times of emotional difficulty, ultimately maximizing the potential of digital therapeutic interventions to support young people’s mental health.

The self-guided aspect of Mello was recognized for offering a nonjudgmental environment, empowering users to address their mental health needs without the pressure that can be felt in face-to-face therapy sessions. Beyond this, it offered users the flexibility to access support and manage their mental health beyond the constraints of traditional clinical hours, particularly when seeking support from family and friends was not feasible or preferred. This adaptability is further explored in theme 2, where participants described Mello as a self-guided tool that offers a sense of control and autonomy over their therapy experience. It provides a nonjudgmental outlet for expression, offers flexibility in therapeutic approaches, and ensures continuous accessibility, ultimately empowering individuals to independently and effectively manage their mental health challenges. This flexibility allows young people to engage, adjust, and switch therapeutic activities in real time, tailoring their experience to their needs and preferences. Thus, although research suggests that dropout rates for self-guided interventions can be as high as 80% [50], the experiences of participants in the Mello trial highlight the significant benefits that self-guided mental health interventions can offer. These include promoting autonomy, flexibility, and reducing anxieties related to therapy. Therefore, understanding how to sustain engagement while maintaining the positive impact of self-guided interventions is crucial for the ongoing development of digital mental health interventions for young people.

### Strengths and Limitations

While all young people assigned to the Mello intervention group were offered the opportunity to participate, it is plausible that those with lower motivation may have been less inclined to participate, potentially skewing our findings. While our themes do capture insights from participants who expressed having low motivation, the results may not fully encapsulate the experiences of this subgroup. This study was also conducted in the context of using Mello within a clinical trial; therefore, the experience of real-world users may be different.

Although members of the youth advisory committee of Orygen provided feedback on the interview questions, the analysis phase lacked direct involvement from a young person with lived experience of mental health challenges. Moving forward, incorporating such perspectives would enrich our research endeavors. Young people with lived experience may identify nuances within experiences that those without such background might overlook, underscoring the importance of their inclusion in the analysis process.

### Conclusions

Through qualitative exploration, our findings identified the value of Mello’s JITAI framework in fostering intentional engagement and reflection on RNT during times of need. This framework reportedly facilitated positive behavioral changes, steering young people away from habitual responses such as resisting support and toward active engagement and reflection on their RNT. These findings align with prior research by Wang and Miller [17], emphasizing the value of tailoring interventions to individuals’ needs and combining human and algorithmic support in digital interventions.

However, some participants faced challenges engaging with Mello due to low mood, stress, and avoidance related to the experience on RNT, which highlights the importance of addressing motivation barriers and avoidance in JITAI. Looking ahead, further research into strategies tailored for individuals with low motivation is imperative. This could involve exploring techniques such as co-design to identify effective personalized support and encouragement as well as furthering the incorporation of user-centered design principles such as personalization and gamification. Such efforts aim to enhance engagement and address the dropout rates associated with self-guided interventions.

Overall, while Mello’s self-guided nature provided a nonjudgmental space and flexibility for users to manage their mental health, sustaining engagement remains a significant challenge. As such, understanding and mitigating these challenges is crucial for maximizing the potential of digital therapeutic interventions to support young people’s mental health in the future.

## Appendix

Multimedia Appendix 1 Description of therapy activities in Mello.

Multimedia Appendix 2 Mello qualitative interview schedule.

## Abbreviations

AI: artificial intelligence
JITAI: just-in-time adaptive intervention
RCT: randomized controlled trial
REDCap: Research Electronic Data Capture
RNT: repetitive negative thinking

## Data Availability

The datasets generated during and analyzed during this study are available from the corresponding author on reasonable request.
