# Supplementary material for: A Personalized, Transdiagnostic Smartphone App (Mello) Targeting Repetitive Negative Thinking for Depression and Anxiety: Qualitative Analysis of Young People’s Experience
Source: J Med Internet Res. 2024 Nov 27;26:e63732. doi: 10.2196/63732 (PMC11635320; doi:10.2196/63732)
Supplement: Multimedia Appendix 2 [file jmir_v26i1e63732_app2.docx]

Appendix B. Mello Qualitative Interview Schedule

1. What was it like to use Mello?
2. What was it like using Mello to address negative thoughts?

Prompt: Were there times when it was helpful?

Were there times when it was not helpful?

1. What was it like getting reminders to check in with Mello?
2. What was it like sharing information with Mello e.g., like how you were feeling and what you were up to?
3. Did you get any feedback from Mello about your stuck thinking? What was that like?
4. Are you able to describe an experience you had with Mello?
5. Could you describe any situations where you found Mello most helpful?
6. Within this study, you had access to Mello for 6 weeks, what are your thoughts on the length of time?

Prompt: too long, too short, just right?

1. Was there anything that kept you using Mello?
2. What sort of things got in the way of using Mello?

Prompt: is there anything the app could have done differently to keep you using it?

1. What might keep other people from using Mello?
2. What about Mello didn’t work well for you?
3. What about Mello worked best for you?
4. Since Mello isn’t finished, what would you like to see in the final version?
5. Could you imagine ways that people like yourself could use Mello with their therapist? Or as part of therapy?
6. What features would help you use Mello in therapy?
